# Optimizing COVID-19 testing resources use with wearable sensors

**DOI:** 10.1371/journal.pdig.0000584

**Published:** 2024-09-05

**Authors:** Giorgio Quer, Arinbjörn Kolbeinsson, Jennifer M. Radin, Luca Foschini, Jay Pandit

**Affiliations:** 1 Scripps Research Translational Institute, La Jolla, California, United States of America; 2 University of Virginia, Charlottesville, Virginia, United States of America; 3 Sage Bionetworks, Seattle, Washington, United States of America; Reader in Forensic Intelligent Data Analysis, UNITED KINGDOM OF GREAT BRITAIN AND NORTHERN IRELAND

## Abstract

The timely identification of infectious pre-symptomatic and asymptomatic cases is key towards preventing the spread of a viral illness like COVID-19. Early identification has been done through routine testing programs, which are indeed costly and potentially burdensome for individuals who should be tested with high frequency. A supplemental tool is represented by wearable technology, that can passively monitor and identify individuals at high risk, alerting them to take a test. We designed a Markov chain model and simulated a routine testing and a wearable testing strategy to estimate the number of tests required and the average number of days in which an individual is infectious and undetected. According to our model, with 2 test per month available, we have that the number of infectious and undetected days is 4.1 in the case of routine testing, while it decreases by 46% and 27% with a wearable testing strategy in the presence or absence of self-reported symptoms. The proposed parametric model can be used for different viral illnesses by tuning its parameters. It shows that wearable technology informing a testing strategy can significantly reduce the number of infectious days in which an individuals can spread the virus. With the same number of infectious days, by using wearables we can potentially reduce the number of required tests and the cost of the testing strategy.

## Author summary

The early identification and quarantine of infected individuals are key to limit the diffusion of viral infections like COVID-19, which is responsible for more than 1 million deaths in the United States alone. While routine testing programs have shown to be effective, they are potentially burdensome for individuals who should be tested with high frequency. Wearable technology has been shown to be a supplemental tool for passive early identification of individuals at risk of infection, enabling an earlier alert to test and shortening the time in which they are infectious and undetected. By inviting individuals to take a test only when they have a higher probability of being infected, this strategy can also reduce the number of required tests.

We propose a model to quantify the effects of this strategy in reducing the spread of the virus. The results from our model suggest that the use of wearable technology can significantly improve the way in which we address the challenges imposed to public health by an ongoing viral illness, reducing the required cost and cognitive load to citizens, while limiting the spread of the virus.

## Introduction

Throughout the COVID-19 pandemic, rapid antigen testing supply has periodically been unable to keep up with demand, hindering public health authorities’ response to identify new cases and limit further community transmission. One of the challenges for preventing the spread of a viral illness is timely identification of infectious pre-symptomatic and asymptomatic cases. Robust contact tracing, paired with frequent testing of populations, is one way to identify these cases before they have the opportunity to transmit infection to others. An aggressive way to identify such asymptomatic cases is to enact routine testing programs, already employed by a number of schools and businesses, where individuals were required to test typically 1–2 times per week. Although these programs are effective, they are also costly, time consuming and burdensome to individuals who must be tested at high frequency to identify most infections before they spread. The conservative counterpart to routine testing is to assume that individuals test only after experiencing symptoms. In 2022, each household in the US was allowed to request 3 times a package with 8 free COVID-19 tests from the government via USPS and more from their insurance provider [1]. Strategies to effectively allocate these limited number of tests, predicting the days in which the infection is present and detectable, could significantly decrease the amount of time people are unknowingly spreading the disease, while minimizing their cognitive and economic burden.

In this study we evaluate the potential of wearable sensor technology [2] as a supplemental health tool to identify an optimal testing strategy [3]. Subtle deviations with respect to baseline have been shown to correlate with the onset of a viral infection and can help identify COVID-19 also in the absence of symptoms [2,4–11], activating individuals towards awareness of their infection and ultimately confirmatory viral testing. In this work, under the assumption that these systems based on wearable sensors can track an infection with a given accuracy, we evaluate how triggering for testing can reduce the total number of tests needed per individual while decreasing the number of infectious days for an infected individual, potentially reducing the spread of the disease. We make our model openly available, allowing the research community and decision makers to test and use the model extensively by varying the underlying assumptions.

## Results

**Fig 1:**
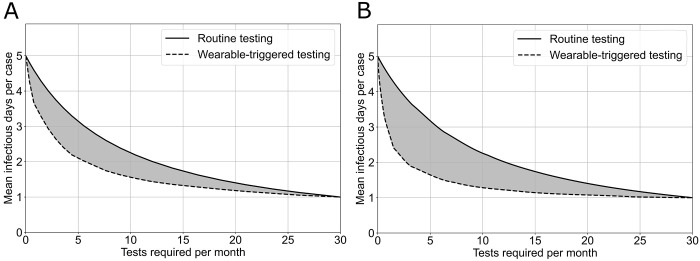
Mean number of undetected infectious days per individuals as a function of the cost expressed in terms of number of tests per month for different testing strategies. (a) in case of wearable testing with sensitivity and specificity described in Fig 2A (no symptoms reported), and (b) in case of wearable testing with sensitivity and specificity described in Fig 2B (cases with symptoms reported).

We specified the available number of tests per month and investigate the performance of the two strategies in terms of number of undetected infectious days (UIDs). A reduction of the UIDs is related to a reduction of the number of individuals that will potentially be in contact to the infected individuals, thus it is related to a reduction in the spread of the disease. In the case of routine testing, if we fix the number of tests per month to 2, according to our model we expect the number of UIDs to be 4.1. Using the wearable sensor trigger strategy, in the absence of self-reported symptoms, the expected number of UIDs is 3.0 (27% decrease in UIDs with respect to routine testing), while in the case with self-reported symptoms it decreases to 2.2 (46% decrease in UIDs with respect to routine testing) (Fig 1).

Alternatively, from a public health perspective, we may need to limit the number of UIDs to contain the spread of a viral disease. From our model, in order to limit the number of UIDs to 2 days, the number of tests needed with the routine testing strategy is 12 tests per month (more than a test every 3 days), while the number of tests needed for the wearable trigger strategy is 2.5 and 5 tests per month, in the presence or in the absence of self-reported symptoms, respectively. (Fig 1). In the presence of symptoms, the reduction in the number of test needed will result in a cost savings of approximately $95 per month per individual (given an approximate cost of $10 per test and 9.5 tests saved per month with the wearable strategy), keeping the number of UIDs to the same level.

The proposed model allows the study of different scenarios by changing input parameters describing the virus pathophysiology, detection performance of wearables (with and without symptoms), or assumptions on individuals behavior. A visual interface (Streamlit widget) on the model parameters is available at https://huggingface.co/spaces/arinbjorn/markov, allowing researchers and decision makers to explore several scenarios and understand tradeoffs between cost and speed of detection.

## Discussion

Our results suggest that incorporating wearables to inform a testing strategy can decrease the number of tests required while minimizing the number of days an individual is at risk of exposing others. The model is a parametric model that can be tuned to different characteristics of the viral illness under examination, like the probability of developing symptoms or the length of time an individual remains infectious. Although encouraging, these results are based on a choice of parameters derived from the delta wave of the COVID-19 pandemic and should be re-estimated for new COVID-19 variants, changes in infectiousness from vaccination, and prevalence of infection. Parameters in our model can be adapted to fit an ever-changing pandemic or even used for other viral illnesses in the future.

Our work builds on our proposed algorithms for detection of COVID-19 infections based on wearable sensor data in the DETECT study [2,9] and on previous prediction models to inform testing frequency [12–14]. Differently from these prediction models, we investigate the incorporation of wearables to predict a COVID-19 infection and inform our testing strategy. In other works, wearable sensors have been used for COVID-19 detection, and even included positivity rate rationale [3]. Building from that work, in order to estimate public-health-relevant outcomes, we simulate the dynamic of infection and detection as compared to standard of care as a function of different testing strategies.

As the SARS-CoV2 virus continues to evolve into new variants that escape immunity, there is still a persistent need to identify strategies that allow early detection of the virus to limit transmissibility and balance that with socio-economic impacts [15]. This is the role of public health screening strategies, that try to minimize time of infectiousness in the most cost-efficient manner, but have not taken advantage of power of digital health tools like wearable sensors [16,17]. Our findings show that with the availability of 2 tests per month, a routine testing strategy will be able to limit the number of infectious days to 4 on average, while our wearable testing strategy will limit the number of infectious days to 2 or 3, depending on the individual reporting or not reporting symptoms. With a routine testing strategy, which is known to be difficult to implement, to limit the number of infectious days to 2, an average of 12 tests per month will be needed while the number of tests needed for the wearable trigger strategy is between 2.5 and 5 tests per month. In order to quickly adapt to new characteristics of the virus we make the model publicly available and provide a visual interface that allows experimenting with new transmission and detection parameters.

Furthermore, each COVID-19 test performed has a personal cost, in terms of time and discomfort in performing the test, and a monetary cost, which varies significantly for different countries, ranging from $1.5 in Thailand to $24 in South Africa, with Germany having one free test per week. (Table A in S1 File) By taking testing costs into consideration, we can come up with an estimated cost savings by implementing a wearable-triggered strategy to optimize testing.

A wearable-triggered testing strategy such as the one described represents an important step towards the direct-to-individual approach that is now possible thanks to digital technologies, the use of advanced AI algorithms to process large amounts of data, and the ubiquitous connectivity that allows us to be in constant contact with individuals [18,19]. At the same time, more personalized public health interventions hinge on increased participation from individuals, whose data is being used to tailor personalized interventions. Individuals using the system should have an informed understanding of what their data is used for, where it is processed (ideally, on-device via edge computing), and be able to revoke that permission at any time. Additionally, since the effectiveness of the system depends directly on individuals responding to the alerts [20,21], trust and engagement with the strategy need to be designed for, not assumed. Participatory design should be used to develop the companion smartphone app to make sure individuals perceive the shared benefits of the intervention, and understand the potential risk for privacy that drove most contact tracing app to failure [22].

Several limitations and unknown—first and foremost behavioral and social dynamics ones—require prospective validation studies to test the proposed strategies and assess their real-world utility by integrating the model into a smartphone app for everyday use. While a strategy using a wearable system to alert about a potential infection has been prospectively tested and its limitations have been highlighted, more research is needed to increase adherence to such system [23]. Furthermore, as with any modeling study, our results are limited to the assumptions we have made. The parameters are tuned to the case of COVID-19 pandemic and in particular the delta wave that spread in 2021, using data collected from our prospective study [9], while the current COVID-19 virus may present different characteristics. However, the proposed model can be quickly adapted to any scenario, including non-COVID-19 infections, by changing its parameters based on new virus characteristics. In our case, sensitivity and specificity of the algorithm have been sourced from a previous clinical trial [9], while results may be different with parameters from other clinical trials run in a different phase of the pandemic. Even in this case, these new parameters can be integrated into our model. We note also that a proper comparison of the strategies and their impact would require a real-world economic analysis, that can better highlight their true costs and benefits. Furthermore, in a future prospective study, also the social cost of potentially over-testing should be properly kept into account. In this work, we assigned a fixed cost for testing, that could potentially include both its economic cost and its social cost.

In addition, we recognize that the use of wearable technology itself has several limitations. First, these algorithms are dependent on how often these devices are worn, since they require almost continuous wearing for best performance. Second, what is known about performance of respiratory illness detection from wearables comes from retrospective studies, which may lead to reporting inflated performance that does not generalize to prospective settings [24] or relies on data requiring real-time collection that may not be technologically feasible. Furthermore, antigen tests that are used in this study have a false negative rate, which may potentially provide an additional source of error for both the routine strategy and the wearable-triggered strategy. Perhaps most importantly, despite the growing use of wearable sensors, there are still challenges to address to mitigate health inequities caused by the digital divide, providing equitable access to wearables [25]. While subsidizing wearable devices may partially address this issue, several challenges in adoption of this technology in a population with low socio-economical status persist. Indeed, while $100 cost per device may seem prohibitive, the significant reduction in number of tests required to limit the spread of the pandemic likely makes this investment in devices cost beneficial. Additionally, 1 in 5 Americans already wear a smartwatch or fitness tracker, making the device investment not necessary for a large portion of the population. Finally, it is important to provide all participants with a clear informed consent before the collection of any data for the implementation of the proposed strategies, and to regulate the access to this data limiting it to the purposes highlighted in the informed consent.

The results from this model suggest that the use of wearable technology, after proper prospective validation, can improve the way in which we address the challenges imposed to public health by an ongoing viral illness, potentially reducing the required cost and cognitive load to citizens, while limiting the spread of the virus.

## Materials and methods

### Ethics statement

All individuals participating in the DETECT study [2] provided informed consent electronically. The protocol for this study was reviewed and approved by the Scripps Office for the Protection of Research Subjects (IRB 20–7531).

### Model for estimating the number of days of undetected infection

In this study, we simulate 2 different testing strategies an individual can employ:

- Routine testing: in this scenario, an individual is tested in the absence of a sensor trigger, with a fixed average number of tests per month that are used at random time intervals (frequency from 1 to 30). In this scenario individuals may still be triggered to test based on their symptoms, exposure, or other scenarios.
- Wearable-triggered testing: an individual is tested when the algorithm triggers an alert to the individual, inviting the individual to get tested.

We develop a model to estimate, for each individual, the number of days an infection remains undetected, potentially spreading the disease. Based on epidemic and wearable detection parameters, we analyze the tradeoff between minimizing the number of days with undetected infection and the number of viral tests required per individual. We perform sensitivity analysis on parameters that are subjected to change as the virus evolves.

**Fig 2:**
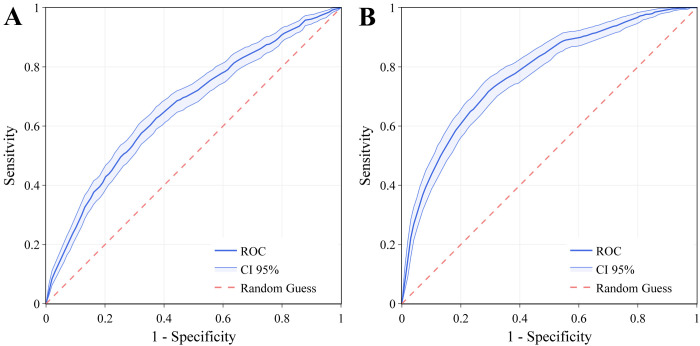
Receiver operating characteristic curves (ROCs) for the discrimination between COVID-19 positive and COVID-19 negative. Performance in the case of no symptoms reported (Fig 2A), and for symptoms reported (Fig 2B). Error bars represent 95% CIs. The figure is adapted from Gadaleta et al., 2021, NPJ Digital Medicine [9], https://creativecommons.org/licenses/by/4.0/.

The wearable-triggered strategy depends on the accuracy of the classifier-based trigger, in terms of sensitivity and specificity. We have used the values specified in the receiver operating characteristic (ROC) curve for a gradient boosting decision tree classifier, trained with more than 8000 individuals in our previous work [9] (Fig 2). In the current work, we consider two scenarios of interest, both considering an epidemic period. The first scenario works for individuals passively using the wearable sensor, who are not reporting any symptoms. This scenario also includes asymptomatic cases. The second scenario considers individuals wearing the sensor and reporting symptoms through a smartphone app.

For both scenarios, we can vary the decision threshold of the classifier, obtaining any pair of sensitivity and specificity represented in the ROC curve. The main assumptions in the choice of the used model parameters have been summarized (Table 1).

**Table 1:** Assumptions at the basis of the model to calculate the number of undetected infectious days for an individual.

Description	Parameter	Assumption and derivation
Probability (daily) of remaining infectious asymptomatic	p_1,1_ = 3/4(1- τ)	The mean number of days in state Infectious Asymptomatic is 4. Thus, according to the memoryless property of the Markov chain, 4 = 1 / (1- p_1,1_), thus p_1,1_ = 3/4. In the case of a testing strategy, this gets scaled by the testing effectiveness, τ.
Probability (daily) of developing symptoms for an infectious asymptomatic	p_1,2_ = 1/8(1- τ)	50% of the individuals who are infectious will become symptomatic at some point in the future.
Probability (daily) of being quarantined for an infectious asymptomatic	p_1,3_ = τ	This is the dependent parameter of the model, which depends directly on the testing strategy.
Probability (daily) of becoming healthy for an infectious asymptomatic	p_1,4_ = 1/8(1- τ)	50% of the individuals who are infectious will become symptomatic at some point in the future. p_1,4_ = p_1,2_ = 1/8(1- τ).
Probability (daily) of becoming asymptomatic (before being detected) for an infectious asymptomatic	p_2,1_ = 0	We assume that everyone who has symptoms is identified and quarantined.
Probability (daily) of remaining infectious symptomatic	p_2,2_ = 1/2(1- τ)	The mean number of days in state Infectious Symptomatic m_2_ = 2 days (on average, an individual is identified and quarantined by the day after becoming symptomatic). According to the memoryless property of the Markov chain, 2 = 1 / (1- p_2,2_), thus p_2,2_ = 1/2. In the case of a testing strategy with effectiveness τ, the probability is scaled with τ.
Probability (daily) of being quarantined for infectious symptomatic	p_2,3_ = 1/2(1+ τ)	Since p_2,3_ = 1 - (p_2,1_ + p_2,2_ + p_2,4_). This is also dependent on testing strategy effectiveness τ.
Probability (daily) of becoming healthy for infectious symptomatic	p_2,4_ = 0	We assume that everyone who has symptoms is identified, thanks to at-home tests.

The technical details in the derivation of the two models, together with the description of hybrid strategies involving both routine and wearable-triggered testing, are detailed in S1 File.

The outcome of the proposed model is the average number of days in which an individual is infectious and undetected, before testing positive. This is a key quantity, as it is proportional to the probability of spreading the virus to other individuals.

## Supporting information

S1 File Supplemental Methods. (PDF)
