# Supplementary material for: Optimizing COVID-19 testing resources use with wearable sensors
Source: PLOS Digit Health. 2024 Sep 5;3(9):e0000584. doi: 10.1371/journal.pdig.0000584 (PMC11376555; doi:10.1371/journal.pdig.0000584)
Supplement: S1 File — (PDF) [file pdig.0000584.s001.pdf]

SUPPLEMENTAL METHODS

In our model, we estimate the number of days in which an individual that became infected remains in an infectious state before one of these two events happens: the individual receives a positive test, thus self-quarantines, or the individual gets healthy and is no longer infectious. The number of days during which an individual is infectious - before the infection is detected - will determine how quickly the virus can spread.

The main assumptions at the basis of our model are listed in the following. 1) We assume that an individual has access to at-home COVID-19 tests when prompted by the wearable system (this assumption is based on the current availability of tests in the US) and will perform the test when prompted. 2) If an individual is asymptomatic, there is still a certain probability of being tested and detect the virus even in the absence of a wearable system for early detection. We assume that an individual is tested on average every N days where N is a parameter of our model. 3) If an individual is symptomatic, they will self-quarantine with a high probability. 4) In case an individual is infected by the virus but has a too low viral load (not infectious), this individual is not considered by our model. In this case, the infection may likely not be detected in case the individual undergoes an antigen test, nor can they spread the virus. For this reason, our model does not deal with these pre-infectious days.

We model an individual that became infectious with a Markov chain with four states: 1) Infectious Asymptomatic, 2) Infectious Symptomatic, 3) Quarantined and 4) Healthy. In our model, an individual can change state once per day (e.g., from an Infectious Symptomatic state in day 3 to a Quarantined state in day 4). An important property of the Markov chain model is the memoryless property, i.e., the future state of the model depends only on the current state, not on the past states of the individual. While this property does not hold exactly in practice (as for

example the probability of recovering does depend on the length of time someone has been sick, not just by whether they're currently sick), it is a customary assumption in SIR (Susceptible, Infectious, or Recovered) and other epidemiological models.

According to this four states Markov model, when an individual arrives to Quarantined or Healthy state, they are no longer spreading the virus. In our model, both these states are absorbing states. To fully define the model, we should describe the following probabilities:

- $p_{1,1}$: probability (daily) of remaining infectious asymptomatic;
- $p_{1,2}$: probability (daily) of developing symptoms for an infectious asymptomatic;
- $p_{1,3}$: probability (daily) of being tested and quarantined for an infectious asymptomatic;
- $p_{1,4}$: probability (daily) of becoming healthy for an infectious asymptomatic;
- $p_{2,2}$: probability (daily) of remaining infectious symptomatic;
- $p_{2,3}$: probability (daily) of being tested and quarantined for infectious symptomatic;
- $p_{2,4}$: probability (daily) of becoming healthy for infectious symptomatic.

Model for individuals without a wearable sensor system to detect COVID-19

In the absence of a wearable sensor that can provide a trigger for testing, an individual in the Infectious Asymptomatic state can be detected positive only by routine testing. If an individual is being tested on average every N days (uniformly at random), we have that the probability of being detected on any given day while in state Infectious Asymptomatic is $\tau = 1/N$. Thus, $p_{1,3} = \tau$. To a first approximation, we assume that half of the individuals who are infectious will become symptomatic at some point in the future.[1] Thus, we can assume that $p_{1,2} = p_{1,4}$.

In case the infection cannot be detected ($p_{1,3} = \tau = 0$), we assume that the mean number of days in state Infectious Asymptomatic is $m_1(\tau = 0) = 4$. Thus, according to the memoryless property of

the Markov chain, we can write $m_1 = 1 / (1-p_{1,1})$, from which we derive $p_{1,1} = 3/4$, and $p_{1,2} = p_{1,4} = 1/8$. In the case in which there is a non-null probability of detection ($p_{1,3} = \tau > 0$), we assume the same proportions among $p_{1,1}$, $p_{1,2}$ and $p_{1,4}$ hold, even if they need to be rescaled so that $p_{1,1} + p_{1,2} + p_{1,3} + p_{1,4} = 1$, thus $p_{1,1} = 3/4\ (1-\tau)$, and $p_{1,2} = p_{1,4} = 1/8\ (1-\tau)$.

In case an individual is in the state Infectious Symptomatic, we assume that the probability of getting healthy without detecting the virus is very small and we approximate it to $p_{2,4} = 0$. On the other hand, there is a very high probability that the individual will self-quarantine when symptomatic even in the absence of routine tests, so the mean number of days in state Infectious Symptomatic is $m_2(\tau = 0) = 2$. We obtain $p_{2,2} = 1/2$. We assume an individual cannot transition from Infectious Symptomatic to Infectious Asymptomatic state ($p_{2,1} = 0$). Furthermore, given that symptoms persist for several days, the probability of resolving all symptoms before self-quarantining or being tested is very low, and we approximate it to $p_{2,4} \simeq 0$. Then $p_{2,3} = 1/2$. In case there is a non-null probability of detection due to routine test ($\tau > 0$), $p_{2,2} = 1/2\ (1-\tau)$ and $p_{2,3} = 1/2(1+\tau)$.

The states Quarantined and Healthy are two absorbing states, for which we have $p_{3,3} = p_{4,4} = 1$. The transition matrix for individuals in the absence of a wearable sensor can thus be written as

$$P = \begin{bmatrix} \frac{3}{4}(1-\tau) & \frac{1}{8}(1-\tau) & \tau & \frac{1}{8}(1-\tau) \\ 0 & 1/2(1-\tau) & 1/2(1+\tau) & 0 \\ 0 & 0 & 1 & 0 \\ 0 & 0 & 0 & 1 \end{bmatrix}$$

The average number of days spent in state Infectious Asymptomatic is $m_1 = 1 / (1-p_{1,1}) = 1 / (1-3/4(1-\tau)) = 4/(1+3\tau)$, while the average number of days spent in state Infectious Symptomatic is $m_2 = 1 / (1-p_{2,2}) = 2/(1+\tau)$. Since the probability of arriving to state 2 (Infectious Symptomatic) is

equal to $P_2 = p_{1,2} / (p_{1,2} + p_{1,3} + p_{1,4}) = 1/2\ (1-\tau) / (1 + 3\tau)$, then we have that the average number of days spent in an infectious state is $m_1 + P_2\, m_2 = (5+3\tau) / [(1 + 3\tau)(1+ \tau)]$.

Model for individuals with a wearable sensor system to detect COVID-19

In the presence of a wearable system for detection of COVID-19, the probability of identifying an individual in state Infectious Asymptomatic depends on the probability of detection by routine tests, τ, and by the probability of detection by the wearable system, π. The probability of detection corresponds to the probability of transition to state Quarantined, and can be written as $p_{1,3} = 1- (1-\tau)(1- \pi)$. The other transition probabilities from state Infectious Asymptomatic, also in this case, are renormalized accordingly such that $p_{1,1} + p_{1,2} + p_{1,3} + p_{1,4} = 1$, thus $p_{1,1} = 3/4\ (1-\tau)(1- \pi)$, and $p_{1,2} = p_{1,4} = 1/8\ (1- \tau)(1- \pi)$.

We assume that, in an Infectious Symptomatic, the same probability π of identification of a positive case holds. In this case, the probability of remaining in Infectious Symptomatic is the probability that the infection is not detected due to symptoms, nor due to routine test, nor due to the wearable system, thus $p_{2,2} = 1/2(1- \tau)(1-\pi)$. From this probability we derive the probability of transition from an Infectious Symptomatic state to a Quarantined state, which is $p_{2,3} = 1/2\ (1 + \tau + \pi - \tau\pi)$. The transition matrix for individuals in the presence of a wearable sensor can thus be written as

$$P = \begin{bmatrix} \frac{3}{4}(1-\tau)(1-\pi) & \frac{1}{8}(1-\tau)(1-\pi) & 1-(1-\tau)(1-\pi) & \frac{1}{8}(1-\tau)(1-\pi) \\ 0 & 1/2(1-\tau)(1-\pi) & 1/2(1+\tau+\pi-\tau\pi) & 0 \\ 0 & 0 & 1 & 0 \\ 0 & 0 & 0 & 1 \end{bmatrix}$$

This is equivalent to the previous transition matrix in the absence of a wearable system, where the probability of detection in the Infectious Asymptomatic case was previously τ. In the presence of a wearable system this probability became $\alpha = 1 - (1- \tau)\ (1- \pi) = \tau + \pi - \tau\pi$.

In the presence of a wearable system, the average number of days spent in state Infectious Asymptomatic is $m_1 = 1 / (1-p_{1,1}) = 4 / [1+3\ (\tau+ \pi- \tau\pi)] = 4/(1+3\ \alpha)$, while the average number of days spent in state Infectious Symptomatic is $m_2 = 1 / (1-p_{2,2}) = 2/(1+\tau+ \pi- \tau\pi) = 2/(1+ \alpha)$. Since the probability of arriving to state 2 (Infectious Symptomatic) is equal to $P_2 = p_{1,2} / (p_{1,2} + p_{1,3} + p_{1,4}) = 1/2\ (1- \alpha) / (1 + 3\ \alpha) = 1/2\ (1- \tau - \pi + \tau\pi) / [1 + 3(\tau + \pi - \tau\pi)]$, then we have that the average number of days spent in an infectious state is $m_1 + P_2\, m_2 = (5+3\alpha) / [(1 + 3\alpha)(1+ \alpha)]= [5+3(\tau + \pi - \tau\pi)] / [(1 + 3\tau + 3\pi - 3\tau\pi)(1+ \tau + \pi - \tau\pi)]$.

Quantify total testing cost by factoring sensitivity and specificity of the wearable technology

In the hybrid testing setting we account for the cost of testing due to both routine testing and when triggered by a wearable. From wearable triggers, we assume that the cost of testing will be dominated by false-positive cases, as opposed to true-positives, given the low prevalence of the disease. To quantify the cost of wearable triggers, we must assume the trigger's specificity. Once the given level of sensitivity is fixed, we obtain the specificity directly from the ROC curves from our previous study.[2]

We also assume that no one will be tested twice on the same day so that in any period of N days there will be a maximum of N tests. With this in mind, and given that the routine testing and wearable triggers are independent we can say that the probability of not being tested on a given day is $(1-p_{routine})*(1-p_{wearable})$, where $p_{wearable}$ is the false-positive rate of the wearable system.

Therefore, the probability of performing any test on a given day is the complementary, $p_{test} = 1-(1-p_{routine})*(1-p_{wearable})$.

Table A. with cost per test in different parts of the world (and different tests). Source for country cost [https://www.theguardian.com/world/2022/feb/11/how-much-does-a-covid-test-cost-around-the-world]. They are rapidly evolving.

Australia	$14.50 -$20
Belgium	$6.50 - $8.50
France	$4.50 - $5.50
Germany	1 free test per week (total government cost $4B from March to October 2021)
South Africa	$24
Spain	$3.20 (free tests available in some regions)
Thailand	$1.50
US	$15
Wholesale [https://www.unicef.org/supply/stories/most-affordable-covid-19-rapid-diagnostic-test-now-available]	*$2.55*

```
import streamlit as st
import altair as alt
import numpy as np
import pandas as pd
st.markdown(
"""
<style>
@font-face {
font-family: 'Tangerine';
font-style: normal;
font-weight: 400;
src: url(https://fonts.gstatic.com/s/tangerine/v12/IurY6Y5j_oScZZow4VOxCZZM.woff2)
format('woff2');
unicode-range: U+0000-00FF, U+0131, U+0152-0153, U+02BB-02BC, U+02C6, U+02DA,
U+02DC, U+2000-206F, U+2074, U+20AC, U+2122, U+2191, U+2193, U+2212, U+2215, U+FEFF,
U+FFFD;
}
html, body, [class*="css"] {
font-family: 'Public Sans', sans-serif;
# font-size: 1rem;
}
</style>
""",
unsafe_allow_html=True,
)
# Define params
st.subheader("Configuration")
col1, col2 = st.columns(2)
# Chances of developing symptoms (per day)
with col1:
symptoms_chance = st.slider(
'Chances of developing symptoms if infected (per day)', min_value=0.0,
max_value=1.0, value=0.5, step=0.01)
# Days spent inf asympt
with col1:
mean_days_inf_asympt = st.slider(
'Mean number of days as infectious asymptomatic (without routine testing)',
min_value=1, max_value=14, value=4, step=1)
base_p00 = 1-(1/mean_days_inf_asympt)
base_p01 = (1-symptoms_chance)*(1/mean_days_inf_asympt)
base_p03 = (symptoms_chance)*(1/mean_days_inf_asympt)
# Days spent inf asympt
with col2:
mean_days_inf_sympt = st.slider(
'Mean number of days as infectious symptomatic (when testing on symptoms only)',
min_value=1, max_value=14, value=2, step=1)
base_p11 = 1-(1/mean_days_inf_sympt)
base_p12 = (1/mean_days_inf_sympt)
# Wearable efficiency
efficiency = st.radio(
"Performance of device",
('Standard', 'Conservative'))
```

```
# Cost case:
# standard case = case in which symptoms are reported if present
sens_list_standard = {0.0: 0.0,
0.005: 0.05,
0.014: 0.1,
0.021: 0.15,
0.05: 0.295,
0.1: 0.434,
0.2: 0.6,
0.3: 0.72,
0.4: 0.79,
0.5: 0.86,
0.6: 0.9,
0.7: 0.925,
0.8: 0.97,
0.9: 0.99,
1.0: 1.0}
# conservative case = symptoms are not reported (it includes asymptomatic cases)
sens_list_conservative = {
0: 0,
0.012: 0.050,
0.026: 0.105,
0.049: 0.149,
0.072: 0.198,
0.096: 0.248,
0.120: 0.297,
0.146: 0.347,
0.184: 0.396,
0.222: 0.446,
0.255: 0.495,
0.300: 0.545,
0.349: 0.594,
0.401: 0.644,
0.467: 0.693,
0.547: 0.743,
0.621: 0.792,
0.699: 0.842,
0.787: 0.891,
0.868: 0.941,
1: 1
}
if efficiency == 'Standard':
sens_list = sens_list_standard
else:
sens_list = sens_list_conservative
def roc_func(x):
return sens_list[x]
def roc_random(x):
return x
# Calculate
test_efficiency = np.array([7, 30, 10000])
FPR = list(sens_list.keys())
days_inf = np.zeros((len(test_efficiency), len(FPR)))
temp_df = []
for tau_count, t_e in enumerate(test_efficiency):
```

```
tau = 1/t_e
for fi_count, fi in enumerate(FPR):
pi = roc_func(fi)
# Transition matrix
p = np.array([
[base_p00*(1-tau)*(1-pi), base_p01*(1-tau) *
(1-pi), 1-(1-tau)*(1-pi), base_p03*(1-tau)*(1-pi)],
[0, base_p11*(1-tau)*(1-pi), base_p12*(1+tau+pi-tau*pi), 0.0],
[0, 0, 1.0, 0.0],
[0, 0, 0.0, 1.0]
])
m1 = 1/(1-p[0, 0])
m2 = 1/(1-p[1, 1])
p2 = p[0, 1]/(p[0, 1]+p[0, 2]+p[0, 3])
days_inf[int(tau_count), int(fi_count)] = m1 + p2*m2
routine_tests_required = 30 * days_inf[2]
# print(routine_tests_required)
# No wearable case
no_wearables = []
tau = 1/10000
for fi_count, fi in enumerate(FPR):
pi = roc_random(fi)
# Transition matrix
p = np.array([
[base_p00*(1-tau)*(1-pi), base_p01*(1-tau) *
(1-pi), 1-(1-tau)*(1-pi), base_p03*(1-tau)*(1-pi)],
[0, base_p11*(1-tau)*(1-pi), base_p12*(1+tau+pi-tau*pi), 0.0],
[0, 0, 1.0, 0.0],
[0, 0, 0.0, 1.0]
])
m1 = 1/(1-p[0, 0])
m2 = 1/(1-p[1, 1])
p2 = p[0, 1]/(p[0, 1]+p[0, 2]+p[0, 3])
no_wearables.append(m1 + p2*m2)
cost = np.array(FPR)*30
no_wearable_cost = cost
# for i in range(len(test_efficiency)):0
wearable_cost = (1-(1-np.array(FPR))*(1-1/test_efficiency[2]))*30
wearable_days_inf = days_inf[2]
# Create chart
chart_data = pd.DataFrame(
{'Tests required per month': no_wearable_cost,
'Routine testing': no_wearables,
'Wearable-triggered testing': wearable_days_inf})
# st.line_chart(chart_data)
chart_data_melted = chart_data.melt('Tests required per month')
print(chart_data_melted)
chart = (
alt.Chart(
data=chart_data_melted,
title="",
height=400,
)
.mark_line()
.encode(
```

```
x='Tests required per month',
y=alt.Y('value:Q', axis=alt.Axis(title='Average case infectious days')),
# y='value:Q',
color='variable:N',
strokeWidth=alt.value(6)
)
.configure_axis(
labelFontSize=20,
titleFontSize=20
)
)
st.subheader("Outcome")
st.altair_chart(chart, use_container_width=True)
```

REFERENCES

1. Ma Q, Liu J, Liu Q, Kang L, Liu R, Jing W, et al. Global Percentage of Asymptomatic SARS-CoV-2 Infections Among the Tested Population and Individuals With Confirmed COVID-19 Diagnosis: A Systematic Review and Meta-analysis. JAMA Network Open. 2021;4(12):e2137257-e.

2. Gadaleta M, Radin JM, Baca-Motes K, Ramos E, Kheterpal V, Topol EJ, et al. Passive detection of COVID-19 with wearable sensors and explainable machine learning algorithms. NPJ Digit Med. 2021;4(1):166.
